# Modeling Dynamic Responses to COVID-19 Epidemics: A Case Study in Thailand

**DOI:** 10.3390/tropicalmed7100303

**Published:** 2022-10-16

**Authors:** Klot Patanarapeelert, Wuttinant Songprasert, Nichaphat Patanarapeelert

**Affiliations:** 1 Department of Mathematics, Faculty of Science, Silpakorn University, Nakhon Pathom 73000, Thailand; 2 Department of Mathematics, Faculty of Applied Science, King Mongkut’s University of Technology North Bangkok, Bangkok 10800, Thailand

**Keywords:** COVID-19, mathematical modeling, control measure, Thailand

## Abstract

Quantifying the effects of control measures during the emergence and recurrence of SARS-CoV-2 poses a challenge to understanding the dynamic responses in terms of effectiveness and the population’s reaction. This study aims to estimate and compare the non-pharmaceutical interventions applied in the first and second outbreaks of COVID-19 in Thailand. We formulated a dynamic model of transmission and control. For each outbreak, the time interval was divided into subintervals characterized by epidemic events. We used daily case report data to estimate the transmission rates, the quarantine rate, and its efficiency by the maximum likelihood method. The duration-specific control reproduction numbers were calculated. The model predicts that the reproduction number dropped by about 91% after the nationwide lockdown in the first wave. In the second wave, after a high number of cases had been reported, the reproduction number decreased to about 80% in the next phase, but the spread continued. The estimated value was below the threshold in the last phase. For both waves, successful control was mainly induced by decreased transmission rate, while the explicit quarantine measure showed less effectiveness. The relatively weak control measure estimated by the model may have implications for economic impact and the adaptation of people.

## 1. Introduction

Infection by the severe acute respiratory syndrome coronavirus-2 (SARS-CoV-2) was first detected in Wuhan, China in December 2019 [1]. The disease was subsequently declared as a pandemic on March 11, 2020, with the name COVID-19 [2]. The deployment of vaccines is expected to withstand the current global pandemic, though nonpharmaceutical interventions are still required for emergencies and uncertainty due to the virus mutations. Several underlying factors are linked, such as the differences in resources, timely responses, economic impact, and social interaction [3,4]. In addition to cross-sectional descriptive study [5], the mathematical model can enlarge the capability to quantify the effects of control measures during the emergence and resurgence of the epidemics.

Thailand accomplished containing the spread of COVID-19 during the first six months of the pandemic. Unfortunately, after no significant domestic infections for almost five months, the country has suffered from multiple infected clusters nationwide since the end of the year 2020 and following another outbreak after the Songkran festival, 12–15 April 2021. The subsequent outbreak was considered as the third wave, which rapidly emerged while the recent second wave had been suppressed around the end of February. Little is known about why the aspect of the new outbreak is different from when the first wave hit in terms of time span, the number of infected people, and the spatial propagation [6].

Several modeling approaches have been used to simulate the temporal dynamics of COVID-19 and assess the effectiveness of public health control measures in Thailand. Most of the existing models, however, emphasize the first wave, while the transmission dynamics of the second wave and the evaluation of control strategy have been less discussed [3,7,8]. Two difficulties behind this potentially involve insufficient data about the origin of the second wave and the aspect of report rate.

In this study, we aimed to examine how the degree of countermeasures to the second wave and their performance change compared with the first wave by using a mathematical model. The control reproduction number is usually used as an indicator for the strength of transmission under control measures. To gain better insight into the effect of control measures, we derive the control reproduction number analytically and analyze the influence of control measures on its component. We examine whether the result could lead us to a better description, scenario or possibly an improvement. To capture the dynamic responses, the parameters are estimated for each time window and used to interpret and compare the performance of public health countermeasure between two outbreaks. As far as the insufficient data about the origin of the second wave is concerned, we fill such gaps by estimating the number of primary cases for the second wave using a simple deterministic epidemic model. As the number of reported cases may be deceptive if one merely fits it directly with the model, we assumed that the actual incidence is underestimated by some factor. The effectiveness of control measures was estimated for each outbreak based on the parameter values obtained from the best fit between the model and daily report data.

## 2. Materials and Methods

### 2.1. Review of COVID-19 in Thailand

Thailand was the first country to find the first infection outside China. The first confirmed case detected on 12 January 2020 was a 61-year-old Chinese woman from Wuhan, followed by the first local transmission reported on 22 January 2020. Even though sparse, the infections since then were continually reported. The government and the Ministry of Public Health started responding by heightening arrival passenger screening at international airports and any border crossing, including international flight cancellations. The large outbreak virtually began in early March, triggered by the superspreading events at a boxing stadium and drinking venues [9]. The Center for COVID-19 Situation Administration (CCSA) in conjunction with the Department of Disease Control was subsequently established to coordinate the government’s response and communicate to the public about the COVID-19 epidemic. An immediate nationwide lockdown followed by curfew on 3 April 2020 was enforced. As a result, the number of new cases continued to fall and subsequently surmounted the first wave around June–July 2020.

On 17 December 2020, there was a confirmed case detected outside the quarantine facilities in Samut Sakhon province for the first time since the end of the first wave; the province has the second highest number of migrant workers in the country. This case had a history of selling seafood at a market.

On 20 December 2020, 535 new locally transmitted cases were reported, which was the largest number of COVID-19 cases reported in Thailand at that time. The burst of new cases overleaped from the two previous days, which were 16 and 34 cases, respectively, due to the extensive contact tracing and active case findings associated with the outbreak in Samut Sakhon. The outbreak area was confined for investigation until no further reports from the area of the outbreak on 24 December. However, the cases linked to this outbreak continuously were detected in surrounding Samut Sakhon, Bangkok, and other provinces.

Until now, the origin of the outbreak in Samut Sakon is not clear, even though the disease investigation might trace back to the first of December 2020. From 24 December 2020 to January 2021, new clusters of cases in Rayong, Chonburi, and other provinces were serially detected. It is remarked that the control measures released by the CCSA aim at specific areas, and the degree varies across the risk zones rather than large-scale strict measures as in the first wave. The risk areas were defined according to case detection and ranked by different levels of control measures. On 2 January 2021, the CCSA divided risk areas where temporal and partial lockdown measures were applied only to maximum control areas comprising 28 provinces. To cope with the second wave, the central control measures had been adapted to ensure a balance between disease control and the economy and to empower local authorities to determine province-specific COVID-19 response measures to suit their situation.

It is remarked that in late January 2021, several provinces had started to ease lockdown restrictions, including the reopening of businesses, even though the number of new cases had not significantly dropped. The observed trend of infections seem to incline downwardly during the middle of February 2021. It was believed that the situation would be more stable from late February to early March, the time that the vaccination program had launched, but there was a sudden spike of a third outbreak in early April.

### 2.2. Data and Timeframe

Daily confirmed cases from January 2020 to February 2021 were collected from COVID-19 Situation Reports by the Emergency Operation Center, Department of Disease Control of Thailand. We defined the first outbreak starting from 12 January 2020 to 31 May 2020 and the second outbreak starting from 1 December 2020 to 28 February 2021. Hence, two subsets of data were separately analyzed following the two periods.

The termination of the first wave is approximately from the end of June to the beginning of July 2020, the period of no case report for several consecutive weeks. We note that the end of February does not imply the end of the outbreak, despite the epidemic curve in the descending phase. Since we concentrate on nonpharmaceutical intervention, the termination point was selected at the beginning of the vaccine operation. For two datasets, the reported cases were counted only for infections within the country, whereas the imported cases that were reported from government state quarantine were excluded.

### 2.3. Mathematical Model

**Figure 1:**
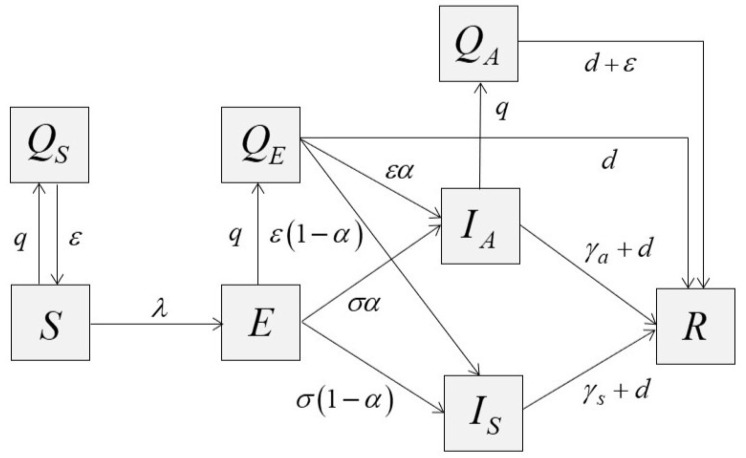
SEIR model with quarantines.

We constructed a deterministic epidemic model to capture the epidemic curves and the performance of control measures. The model’s purpose is to provide the best fit between data and model results. To this end, we adapted the SEIR model by classifying the infectious state into symptomatic ($I_{S}$) and asymptomatic ($I_{A}$) states with the quarantine. We assumed that the number of total population (*N*) is constant, and the exposed class (*E*) is not capable of infecting the others. The recovery state (*R*) does not imply an actual number of recovery, rather, it combines all patients who were isolated, recovered, and died. Figure 1 displays flowing between compartments where the description of model parameters is informed in Table 1. The differential equations of the model was shown in Appendix A.

We assumed that the total number of quarantined people at time *t* is

$$Q\left(t\right)=Q_{S}\left(t\right)+Q_{E}\left(t\right)+Q_{A}\left(t\right).$$

The variable $Q_{i},i\in \left\{S,E,A\right\}$ represents the number of quarantined people in disease state *i*, where *S* stands for the susceptible state, *E* denotes the exposed state, and *A* denotes the asymptomatic state. In this model, the quarantine has wide implications. An individual can be quarantined either under the control of public health authorities while contact tracing or by self-quarantine at home. Thus, quarantined, susceptible individuals may be caused by the effect of movement restriction such as closing public places, including workplaces and schools. An exposed individual can have a negative test result during quarantine. Since the quarantine period for two weeks is probably longer than the incubation period, once it elapses, the exposed individual is going to end up either asymptomatic or symptomatic. We assumed that the effectiveness of isolation is perfect, so symptomatic infections are not quarantined but will undergo isolation, which eventually results in recovery or death.

We assumed that the asymptomatic infection will not be isolated but may be quarantined. Indeed, they may be occasionally tested, and hence isolated. We ignored this fact, since the rate of tests, in general, was very low in Thailand at that time. Once they are quarantined, they can be tested or depart after two weeks. Since the quarantine period is assumed to be longer than the average infectious period, they are moved into the recovery class in any case.

### 2.4. The Reproduction Number

We calculated the so-called *control reproduction number*, $R_{c}$, or in short *reproduction number*, defined as an average of secondary cases through the infectious period initiated by a single case subject to the control measure. By using the next generation method, we find

(1) $$R_{c}=\psi_{s}\psi_{e}\left(\alpha R_{a}\psi_{a}+\left(1-\alpha \right)R_{s}\right),$$

where

$$\begin{array}{ccc} \psi_{s} & = & 1-\frac{\xi q}{q+\epsilon },\\ \psi_{e} & = & 1-\frac{dq}{\left(\epsilon +d)(\sigma +q\right)},\\ \psi_{a} & = & \frac{\gamma_{a}}{q+\gamma_{a}}\left(1+\left(1-\xi \right)\frac{q}{\epsilon +d}\right) \end{array}$$

describe the effect of quarantines, and $R_{a}=c\beta_{s}/\gamma_{a}$ and $R_{s}=\beta_{s}/\gamma_{s}$ describe the transmission potentials of asymptomatic and symptomatic infections in isolation, respectively.

We now explain the effects of quarantine as follows. In the absence of quarantine of susceptible individuals, the susceptibility is unity since the whole population is counted. In the presence of quarantine, the susceptibility is given by $\psi_{s}$, where the reduction is

$$\frac{\xi q}{q+\epsilon }.$$

The parameter $\psi_{e}$ describes the ratio between incidence and the outflow rate of exposed class (see Appendix B). In the absence of case detection, i.e., d=0, we see that $\psi_{e}=1$. It is clear that all exposed individuals who left the quarantine state became either symptomatic or asymptomatic cases. Thus, quarantine of exposed class in this case is almost unaffected but delays the new infections. On the other hand, if d>0, we have, $\psi_{e}<1$, its expression equals one mimus a fraction

$$\frac{dq}{\left(\epsilon +d)(\sigma +q\right)},$$

which implies that $\psi_{e}$ can be viewed as the percentage reduction in the infectivity due to the quarantine of exposed class. Thus, a strict test among quarantine individuals is necessary.

The parameter $\psi_{a}$ describes the mean infectivity of asymptomatic infections. It is a product of the probability that an asymptomatic individual will not be quarantined and the fraction of active asymptomatic individuals. For the latter, the effect of quarantine depends not only on the percentage of detection among quarantine but the effectiveness of quarantine itself, i.e., ξ. This is because the more fractions of quarantine that escape, the more force of infection induced by the asymptomatic cases. The model suggests that increasing quarantine rates can reduce the infectivity of asymptomatic transmission if

$$\xi >1-\frac{\epsilon +d}{\gamma_{a}}.$$

Conversely, we have $\psi_{a}>1$. In the cases that $\psi_{a}<1$, the infectivity is reduced by $1-\psi_{a}$.

### 2.5. Parameter Estimations

We used the maximum likelihood method for fitting the incidence function to the daily reported cases [12]. The parameters to be estimated are the transmission rate of a symptomatic patient, $\beta_{s}$, quarantine rate, *q*, and its efficacy ξ, respectively. These parameters characterize the contact pattern, transmission risk, and effectiveness of control measures, which vary across populations. The test rates were separately estimated from the data by averaging over focusing time period. The rest was extracted from previous studies.

We used Poisson distributions to construct the likelihood function and assumed that the observed incidence is independently distributed with mean m(t). The model was solved numerically to calculate m(t) and the likelihood function. To be more specific, we denote $x_{i}$ as the number of new cases observed at time $t_{i}$, and $\vec{\theta }$ is a vector of parameters to be estimated. The log-likelihood function for Poisson distribution is given by

(2) $$L\left(\vec{\theta }\right)=\sum_{i=0}^{n-1}-m\left(t_{i}\right)+x_{i}\ln m\left(t_{i}\right),$$

where $m(t_{i})$ are calculated from the incidence function, which depends on $\vec{\theta }$. Thus,

(3) $$\hat{\theta }=\arg\max_{\vec{\theta }}L\left(\vec{\theta }\right),$$

gives a solution to the maximum likelihood estimation problem. To calculate the incidence function associated with daily reported cases, we let θ be the fraction of new infections that have been reported. By means of the model, the incidence function can be estimated by

(4) $$m\left(t\right)=\theta \left(1-\alpha \right)\left(\sigma E\left(t\right)+\epsilon Q_{E}\right)+d\left(Q_{E}\left(t\right)+Q_{A}\left(t\right)\right).$$

The first term indicates the new infections that have symptoms, while the second term indicates the new infections found in quarantine.

In the first wave, the outbreak event is composed of three phases. The first phase starts from 12 January 2020 to 5 March 2020. The beginning of the superspreading event on 6 March 2020 is defined as the beginning of the second phase. The third phase begins on 26 March 2020, the day that the government announced a state emergency decree to control COVID-19, resulting in the nationwide enforcement of travel restrictions or lockdown. Soon after the nationwide curfew was announced, we note that the determination of each phase is similar to the description in Triukose et al. [8], especially, the first phase coincides. Our second phase, however, covers the second phase of Triukose et al. and somewhat of their third phase. The beginning of our third phase is the same as defined in Mahikul et al. [7]. This different viewpoint depends on the extent of detail consideration and the size of data required for model fitting.

Similarly, the second wave is composed of three phases. The origin of the second wave is not known. According to the large number of new cases reported on 20 December 2020, we estimated that the early infection of the cluster had possibly started in early December. The beginning phase of the second wave is thus defined from the first of December to 20 December. As the partial lockdown had been applied, several new clusters took place across the regions. We thus choose the day that the highest number of reported cases was seen as the endpoint (26 January 2021). The final phase of the second wave ends on 28 February, the day that the vaccination program launched.

In the first wave, the initial setting was a single symptomatic infectious individual imported from China. The model was run through the third phase, by which the initial condition was calculated at the end of the previous phase.

To estimate the number of primary cases in the second wave, we employed the cumulative number of cases from 1 December 2020 to 20 December 2020, which is 559 cases. Simultaneously, we also estimated the transmission rate within this period by assuming that the other parameters were fixed as analogous to the initial phase of the first wave. We thus estimated parameters by minimizing the difference (error) between the cumulative number of reported cases calculated by the model and 599. The sensitivity of initial guess on the estimation was considered.

## 3. Results

### 3.1. The First Wave

**Figure 2:**
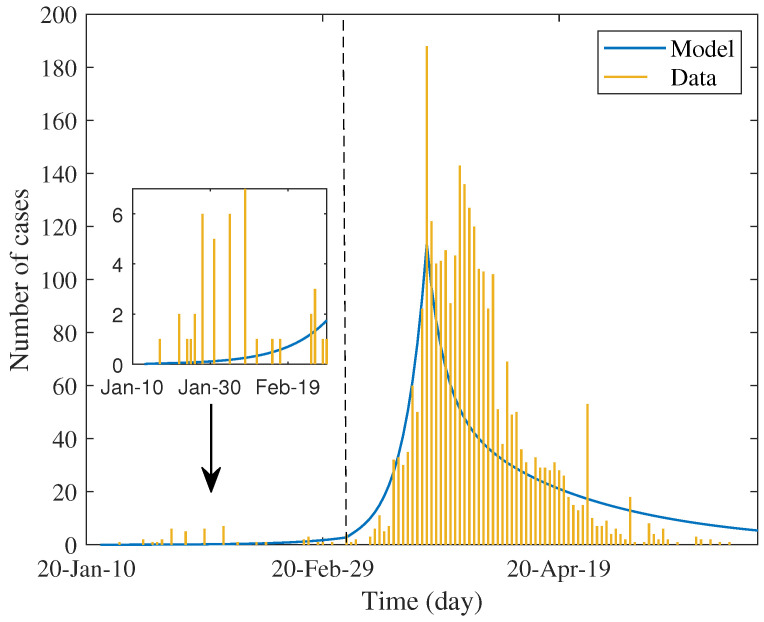
Fitting daily reported cases over a six-month period of the first wave. Small bars represent the data and solid lines present the models. The vertical dashed line indicates the boundary between phase I and II, while the peak indicates the beginning of phase III. Inset shows minor epidemics in phase I.

Table 2 shows the estimated values of parameters and percentage of reduction in each factor. The first three parameters were estimated by maximum likelihood method and the test rate was estimated separately from test data. We used these values to simulate the daily case report, which was assumed to be twenty percent of estimated incidence. The result shown in Figure 2 determines the trend of data in each phase.

In Table 2, the estimated value of the transmission rate lifted in the second phase and dropped in the last phase; meanwhile, the estimated value of the quarantine rate moves from phase to phase in the opposite direction. Prominently, the estimated value of effectiveness of quarantine is highest in the last phase, i.e., about 0.56. The changing pattern of these values asserts what had happened and implies the efficiency of lockdown measures.

In the first phase, the control reproduction number is approximately 2.6, while the basic reproduction number can be calculated as $R_{0}=\alpha R_{a}+\left(1-\alpha \right)R_{s}\approx 2.76$ that was close to the minimum of early estimation, i.e., 2.23–5.9 in [13], but on average 2.47–2.89 in [7]. This showed that the response to the early epidemic yielded a little positive effect by reducing by about 5.8% of the basic reproduction number. Nevertheless, the observed epidemic was *minor* and rarely dispersed (see the inset of Figure 2). As mentioned earlier, in addition to the local contact investigation and the stringent level of screening measures for travelers who arrived, quarantine measures had not been formally and systematically practiced. Most of the self-quarantine is probably linked to the psychological impact of news, resulting in the wide alertness of the population. It is also observed that such quarantine most affects susceptible groups (reducing by 0.19%). Strikingly, the reduction in infectivity of asymptomatic infection is negative. This means that the quality of the quarantine can lead to the reverse effect. This is because the estimated effectiveness, ξ, is lower than the critical condition, hence the reverse effect occurs.

The parameters estimated in the next phase captured the superspreading events related to drinking venues and a boxing stadium. The magnitude of the estimated reproduction number is about two times increased from the early phase. The model predicted that the quarantine rate and its efficacy dropped more than ninety percent from the previous period.

After the nationwide lockdown had started, the observed number of new cases dropped downward with no rebound. Accordingly, the estimated reproduction number decreased by about 91% from the previous phase; its value is less than one. The quarantine rate is approximately similar to the initial phase, but the effect is much better. The model describes that the strict measure had a significant impact on the transmission chain by mitigating the transmissibility and reducing both susceptibility and infectivity. As in the first phase, the strict lockdown measure has the most impact on susceptibility. Nevertheless, the reduction in all factors lifted up with respect to the first phase.

### 3.2. The Second Wave

**Figure 3:**
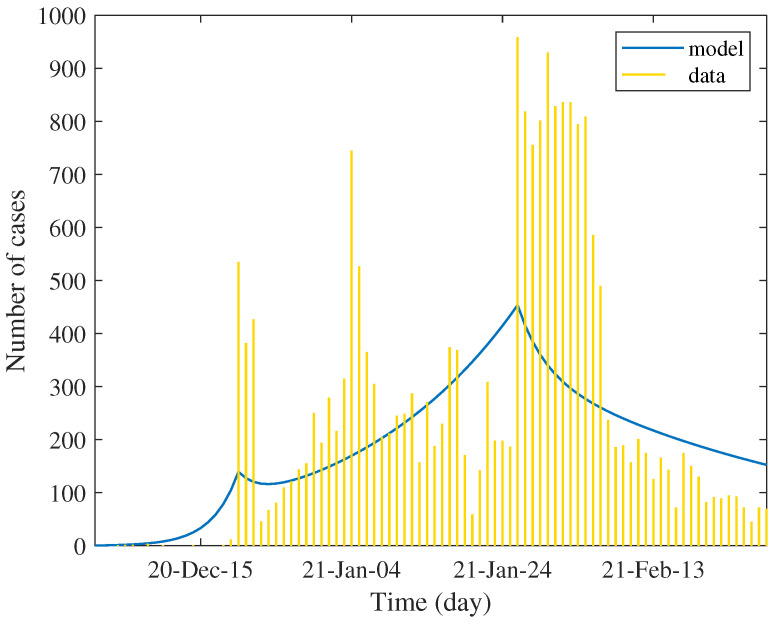
Curve fitting for daily case reports in the second wave. Small bars represent the data and solid lines present the models.

We find that the estimated asymptomatic and symptomatic primary cases were about 1 and 12, respectively, and the estimated transmission rate of symptomatic case is about 1.25 (see Appendix C). Table 3 shows the values of estimated parameters and the percentage of reduction in each factor. We used these values to simulate the daily case report, which was assumed to be twenty percent of estimated incidence. The result shown in Figure 3 determines the trend of data in each phase. Unlike the first wave, the data show several peaks of case reports between defined phases. This is due to the very low rate of testing in the general population; the report was mainly subject to immediate active case finding of emerging clusters. Technically, this creates a difficulty in numerical optimization.

The estimation result shows that the strength of the epidemic in the second wave is much higher than that of the first wave. The estimated number of primary cases and the transmission rate are evident. The control reproduction number was about 3.5 times higher compared with the first phase of the first wave and about 1.6 times higher compared with the second phase of the first wave. As a consequence, the reduction in all components is relatively low since we assumed that the level of quarantine measure is similar to the initial phase of the first wave.

In the second phase, the control measure with population reaction approximately reduces the control reproduction number by about eighty percent. The model shows that this reduction may not be induced by the quarantine but by the transmissibility. The travel restriction might indirectly affect the contact rate, while the face masking, social distancing, and hygiene practices reduce the transmission probability. As seen in the table, the effect of quarantine in this phase is very low. The reverse effect was also shown. Although the reduction in transmission rates is significant, it is not sufficient to depress the reproduction number below unity. The trend of incidence was slowly increased, approaching the peak by spending 37 days. This period covers Christmas to the New Year festival, which is believed to be a factor of unsuccessful quarantine.

In the last phase, the transmission rate dropped by 60% from the previous phase and by 93% from the initial phase. The estimated effectiveness of quarantine appears little improved. Since the most reduction came from the reduced transmission rate, the effect of quarantine in this phase is comparatively low. However, the predicted control reproduction number is less than one, signaling a somewhat effective control measure. The model showed the monotonically decreasing trend of incidence.

### 3.3. Comparing Dynamics of the Scale of Quarantine between Two Waves

**Figure 4:**
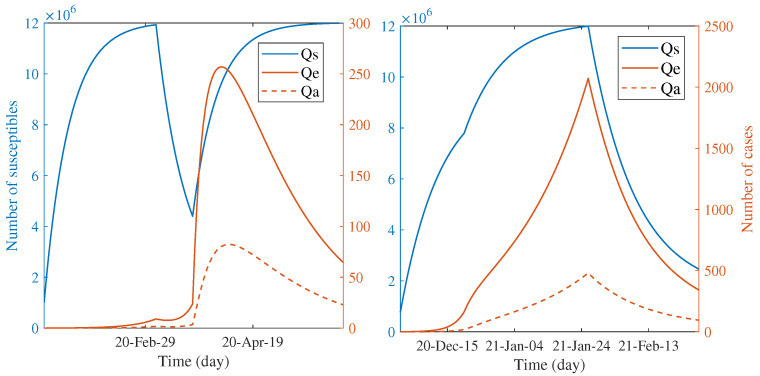
Dynamics of the quarantined people during the epidemic calculated by models. **Left** and **right** figures present the number of susceptible, exposed, and asymptomatic individuals that were quarantined in the first wave and in the second wave, respectively. Both figures are plotted with two different scales on the *y*-axis (colors), where the left (blue) represents the number of susceptible people in quarantine and the right (red) represents the number of infected people in quarantine.

Another way to characterize the impact of countermeasures is speculating the number of quarantined individuals through the course of outbreaks. Figure 4 illustrates the predicted trend of the quarantined people during each wave according to the estimated parameters. For both waves, the number of quarantined, susceptible individuals continually increased during the first phase. The infected individuals had been quarantined a bit after the quarantine of susceptible groups; they are also shown in the smaller scale. The observed dynamic patterns between two waves are distinct when the first phase elapsed. The trend of susceptible individuals declined for the first wave but continually increased for the second wave. This is clear since the countermeasure had been started in the second wave, whereas it had not for the first wave. In the first wave, the turnover of susceptible people to be more quarantined was shown after the announcement of the nationwide lockdown; the number tends towards constant until the end of the epidemic. On the other hand, during the last phase, the quarantine of susceptible people seems to be relaxed in the second wave. The quarantine of infected classes for both waves have the same patterns, excepting for the position and scale of the peaks. Such different patterns between two waves indicate the dynamic difference of responses in terms of the degree of caution in the general population and the effectiveness of control measures.

### 3.4. Probably Improving the Effectiveness of Control Measures

In this section, we seek to find the probable improvement of the quarantine measure based on model results. We learned that, for both waves, the model of quarantine measure either by self-quarantine or by contact tracing shows to be less effective. Rather, the main factors influencing achieved containment were the transmission rates, which are practically difficult to control with the requirement to counterbalance economic impact. This is evident in the second phase of the second wave since the CCSA aimed to define zones and confine the travel-only risk areas rather than mandate a nationwide lockdown. This results in the delay of the epidemic peak, so the more stringent effort required flattening the curve. In accordance with the adjusted policy, testing rate, *d*, and the effectiveness of case finding quarantine, ξ, are essential to foster the quarantine measure. It is worth noting that these two parameters are independent of the level of lockdown. Thus, the recommended strategy should be placed on increasing these two parameter values.

Three possible scenarios are considered as follows: (i) increase ξ alone, (ii) increase *d* alone, and (iii) increase both ξ and *d* together. From Equation (1), the first scenario decreases $\psi_{s}$ and $\psi_{a}$, the second one deceases $\psi_{e}$ and $\psi_{a}$, and the last one decreases all influential factors. Since the quarantine of asymptomatic cases had a reverse effect on reducing the infectivity, the goal is to determine to which extent the control measure improves as the reverse effect vanished. More precisely, we are interested in the improved control measure at the point $\left(d^{*},\xi^{*}\right)$, such that

(5) $$\xi^{*}=1-\frac{\epsilon +d^{*}}{\gamma_{a}}.$$

With this condition, $\psi_{a}=1$. For generalization, the range of variation can be expanded beyond this condition, yet we specified it to avoid unnecessary complexity. Since we concentrated on the second phase of the second wave, the estimated values of (d,ξ) in that phase are given as a baseline. For the first and second scenarios, only a single parameter is determined from Equation (5). For the last scenario, the point $\left(d^{*},\xi^{*}\right)$ is determined by an intersection between the line in Equation (5) and its perpendicular passing through the baseline point in the dξ−plane. We measured the efficiency of the new control measure by calculating the relative change in the control reproduction number with respect to the control effort

(6) $$\text{Efficiency index}={\left(\sum_{i}^{n}\frac{p_{i}^{\left(new\right)}}{p_{i}^{\left(prev\right)}}-n\right)}^{-1}\left(\frac{R_{c}^{\left(new\right)}}{R_{c}^{\left(prev\right)}}-1\right),$$

where $p_{i}$ denotes the parameter of interest and *n* is the number of parameters. The first term of the above expression defines the control effort.

The results shown in Table 4 reveal the three ways of countermeasure improvement subject to a common constraint. By the efficiency index, increasing the effectiveness of quarantine alone gives the best improvement and the highest reduction in $R_{c}$ with relatively minimal control effort. Conversely, increasing the testing rate alone yields the lowest reduction in $R_{c}$ with relatively maximal control effort. The last scenario seems to compromise between the two scenarios. It is noteworthy that, in the last case, the required control effort is comparable to the second case, the change in ξ is small, but the change in *d* is quite large. The idea is, however, flexible, since one can pick the proper testing rate and then calculate ξ from Equation (5). Furthermore, the efficiency is used to evaluate the new control strategy, which is easy to implement.

## 4. Discussion

The dynamic model of the spread of COVID-19 in this study may be comparable to several models emphasized on the effects of quarantine measures. Apart from simplicity, some prominent features of our model that are different from such models should be noticed. First, the dynamic of quarantine for susceptible, exposed, and asymptomatic groups was assumed to be linear, while its effectiveness affects both total susceptibility and the force of infection. This is different from [14,15], such that the quarantine is focused only on the infectious group, and there is no effect on susceptibility, respectively. Second, we assumed that there is no transition between quarantine groups, whereas it was assumed to be possible in [16,17].

Decomposing time into several windows has pros and cons. Single estimation per outbreak may lead to an inappropriate overall description and of course cannot capture dynamic responses during epidemic events. In a retrospective study, the selection of time windows depends on available information and the purpose. Our selection in the first wave slightly differs from previous works [7,8], yet the results are qualitatively similar. However, due to the discreteness, it cannot capture continuous patterns of response which reflect cause and effect between phases in case of insufficient information. For instance, the presence of abruptly reported cases in the second wave makes the influence of control measures quite obscure. The daily case reports present several peaks within the selected time window. This is actually required subdividing. Nevertheless, the selection of time window is restricted to the sufficient data size used in numerical optimization.

As mentioned earlier, the quarantine rate in this study has specified meaning. Every state was assumed to be quarantined with the same rate to keep the estimation algorithm stable, and the number of parameters to be estimated in the first step should be minimal. To be more realistic, the rate can be distinguished by disease state. Thus, the fluctuation of quarantined people here is driven by the dynamic of each disease state. The estimated quarantined rate can be viewed as an average of all states. The change in its estimated value of each phase reflects the qualitative difference in the strength of control measures, psychological impact, and degree of compliance of people.

To our knowledge, our study is the first to model the second wave in Thailand. The model predicts high virulence at the initial stage of the epidemic among the worker community in Samut Sakorn province. According to public health report, the epidemic probably started approximately 20 days before the day of large cases being reported. The variation of that duration might alter the predicted results. The longer period possibly decreases the estimated primary cases or the transmission rates but should not significantly alter the aspect of the predicted incidence curve and the following estimated reproduction number. The moderately inclined reproduction number in subsequent periods indicates the change in government policy from countrywide lockdown to local confinement with contact tracing. The so-called *bubble and sealed measure* was instantly applied after the cluster had been detected. Seemingly, this control measure shows a defect, since the high number of cases was subsequently reported. It is remarked that the observed dissemination of the new clusters may be caused by the limitation of the active case finding in the previous cluster, which possibly does not cover all the cases, including somewhat relaxing travel restrictions. Another possibility is that the time that the first cluster was reported might not indeed be the early phase. In terms of modeling, this may create an elusive pattern that does not reflect the actual transmission. This is why the estimated report rates seem to be underestimated.

The modeling approach in this study has at least three technical limitations. First, the useful analytic formula is the trade-off simplicity. The pre-symptomatic state is one of the missing model components, which is known as playing an additional role in transmission. Incorporating this compartment might alter the parameter estimation, especially weighing the role of asymptomatic infections resulting in reducing the overestimated asymptomatic ratio. Nevertheless, the maximum likelihood estimation can become unstable when the number of parameters increases, since the testing, quarantine, and isolation must be further accounted for at that stage. Second, we specified the Poisson distribution as a statistical model for every estimation. An opportunity to improve the performance of parameter estimation is the test of statistical dispersion [18]. For example, when actual overdispersion is detected in some phase of the epidemic, one may replace Poisson with a negative binomial model that allows for the variance to be greater than the mean. Third, the present dynamic model has difficulty interpreting the impact of specific control measures. It allows only evaluating the impact of overall control measures through the reduction in transmission rates, susceptibility, and infectivity. For instance, a reduction in transmission rates could be induced by either face masks or social distancing. Moreover, the meaning of social distancing, movement restriction, and quarantine can be interchangeable. The quarantine of susceptible persons can take all three meanings. Connecting specific measures to model parameters is challenging to empower evaluation.

The estimated parameters reveal several pieces of information that are unable to be extracted solely by data. We observed that the effect of a quarantine measure on both outbreaks is weak. Although the predicted control reproduction number in the final phase is less than one, the weakened consistency in terms of the policy and population reaction may create a potential risk for the new cluster from this point, excepting if the vaccination is sufficiently covered.

## 5. Conclusions

In this study, we reviewed the epidemiology of COVID-19 from the beginning episode to the starting of the vaccination program in Thailand. We used data as clues together with a mathematical model to examine and compare the effectiveness of control measures applied to two epidemic waves. By dividing the period into a set of subintervals, the reproduction number and contributing parameters were treated as time dependent. The reproduction number was analytically derived, after which its components were used to capture the effect of the control measures. The results show that the epidemic in the first wave had successfully been controlled by stringent and inclusive measures. In the second wave, we estimated the number of primary cases and the transmission rates by using the cumulative number of reported cases. The result reveals thirteen primary cases with only one asymptomatic infection, and the transmission rate of about 1.25 yields the minimum error. They were subsequently used throughout the calculations. From the day that the highest number of cases was reported at the first time, the control measure fails in complete elimination within a suitable period, as in the first wave. Although the factors influencing this are currently debated, we hypothesized that the more relaxed policy should be induced by economic impact and attitude change in society. Under this circumstance, the model results indicate that the effectiveness of quarantine measures should be improved along with increasing testing. We hypothetically recommend the improved strategies that might be appropriate for the change in policy.

Thailand has been experiencing an acute outbreak while the vaccination has been simultaneously operating. As the economic factor is counterbalanced, the stringent nonpharmaceutical measures become temporary or unable to deal with the spread. Nevertheless, the more precise evaluation of control measures is still required for improving management, not only for SARS-CoV-2 but for possible new emerging diseases.

## Figures and Tables

**Table 1:** List of model parameters.

Symbol	Definition	Value	References
$\beta_{s}$	symptomatic transmission rate	estimate	
*c*	ratio of transmissibility between asymptomatic and symptomatic case	0.418	[10]
σ	outflow rate of exposed state	1/5.2 day${}^{-1}$	[7]
α	asymptomatic ratio	0.3	[11]
ξ	effectiveness of quarantine	estimate	
$\gamma_{s}$	recovery rate of symptomatic infection	0.10526 day${}^{-1}$	[7]
$\gamma_{a}$	recovery rate of asymptomatic infection	0.2 day${}^{-1}$	[7]
*d*	the rate at which an infection is detected during quarantine	estimate	
*q*	quarantine rate	estimate	
ϵ	outflow rate of quarantine	1/14 day${}^{-1}$	[7]

**Table 2:** Estimation of parameters, the reproduction numbers, and percentage of reduction for the first wave.

Parameter	Phase I	Phase II	Phase III
$\beta_{s}$	0.379	0.804	0.084
*q*	0.015	0.001	0.015
ξ	0.011	0.001	0.514
*d*	2.60$\times 10^{-6}$	2.10 $\times 10^{-5}$	7.20 $\times 10^{-5}$
$R_{a}$	0.7921	1.6804	0.1756
$R_{s}$	3.6006	7.6382	0.7980
$R_{c}$	2.7821	5.8553	0.5579
Percentage of reduction			
Susceptibility	0.1909	0.0014	8.9207
Infectivity by latency	0.0003	0.0002	0.0073
Infectivity by infectious	−12.3426	−0.8937	−2.5076

**Table 3:** Estimation of parameters, the reproduction numbers, and percentage of reduction for the second wave.

Parameter	Phase I	Phase II	Phase III
$\beta_{s}$	1.25	0.2412	0.0943
*q*	0.015	0.0151	0.0016
ξ	0.011	0.012	0.0122
*d*	7.51$\times 10^{-5}$	2.99$\times 10^{-4}$	2.41$\times 10^{-4}$
$R_{a}$	2.6125	0.5041	0.1971
$R_{s}$	11.8754	2.2915	0.8959
$R_{c}$	9.1748	1.7696	0.6869
Percentage of reduction			
Susceptibility	0.1909	0.2094	0.0267
Infectivity by latency	0.0076	0.0303	0.0028
Infectivity by infectious	−12.3230	−12.3193	−1.3941

**Table 4:** Improvement of quarantine measure by increasing testing rate and the effectiveness of quarantine based on the parameters estimated in the second phase of the second wave.

Parameter	Scenario I	Scenario II	Scenario III
$\xi^{*}$	0.6414	0.0120	0.0362
$d^{*}$	0.0003	0.1262	0.1213
$R_{c}$	1.5583	1.6702	1.6642
Reduced Susceptibility	11.1924	0.2094	0.6318
Reduced Infectivity by latency (%)	0.0303	4.6486	4.5825
Reduced Infectivity by infectious (%)	0.0000	0.0000	0.0000
Control effort	52.4471	421.6849	407.4834
Relative change in Rc	0.1212	0.0582	0.0615
Efficiency index	0.0023	0.0001	0.0002

**Figure A1:**
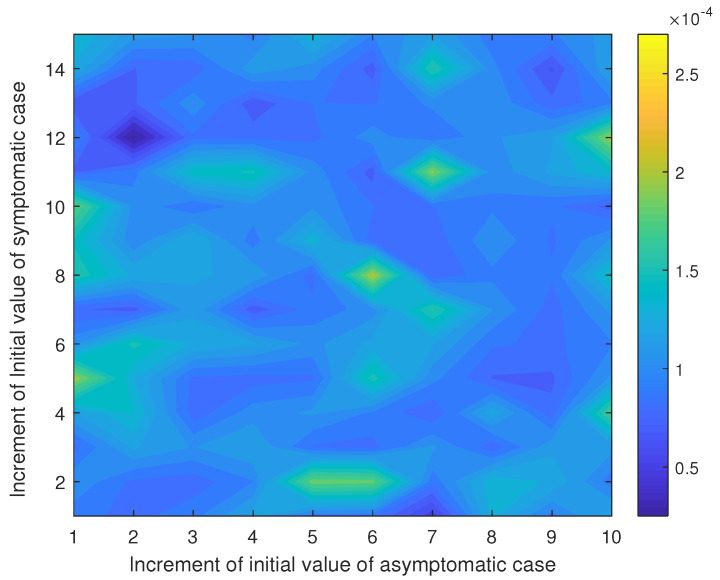
Variation of the starting point for optimization algorithm on the absolute errors of predicted cumulative number of reported cases at the end of the first phase.

**Figure A2:**
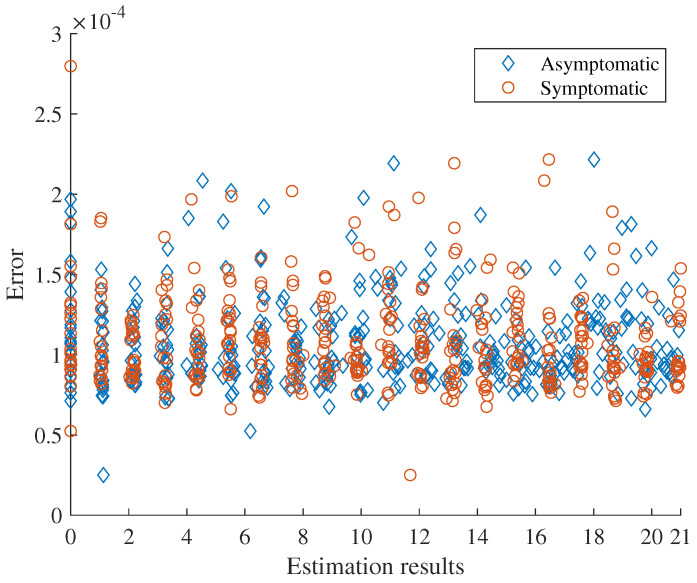
Errors were plotted with respect to the optimization results. The minimum point for asymptomatic case is one, whereas the minimum point for the symptomatic cases is twelve.

## Data Availability

The datasets generated and/or analyzed during the current study are available in the DDC OpenData repository, https://covid19.ddc.moph.go.th/en (accessed on 10 January 2022).

## Abbreviations

The following abbreviations are used in this manuscript:

SARS-CoV-2	Severe acute respiratory syndrome coronavirus-2
COVID-19	Coronavirus disease 2019
CCSA	Center for COVID-19 Situation Administration
SEIR	Susceptible–Exposed–Infectious–Recover

## Appendix A. Mathematical Model

The dynamical equations that describe the model can be expressed as

(A1) $$\begin{array}{ccc} \frac{dS}{dt} & = & -\lambda \left(t\right)\left(S+\left(1-\xi \right)Q_{S}\right)-qS+\epsilon Q_{S},\\ \frac{dE}{dt} & = & \lambda \left(t\right)\left(S+\left(1-\xi \right)Q_{S}\right)-\left(q+\sigma \right)E,\\ \frac{dI_{A}}{dt} & = & \sigma \alpha E+\epsilon \alpha Q_{E}-\left(\gamma_{a}+q\right)I_{A},\\ \frac{dI_{S}}{dt} & = & \left(1-\alpha \right)\left(\sigma E+\epsilon Q_{E}\right)-\gamma_{s}I_{S},\\ \frac{dQ_{S}}{dt} & = & qS-\epsilon Q_{S},\\ \frac{dQ_{E}}{dt} & = & qE-\left(\epsilon +d\right)Q_{E},\\ \frac{dQ_{A}}{dt} & = & qI_{A}-\left(\epsilon +d\right)Q_{A}. \end{array}$$

The force of infection reads

(A2) $$\lambda \left(t\right)=\frac{\beta_{s}}{N}\left(c\left(I_{A}+\left(1-\xi \right)Q_{A}\right)+I_{S}\right).$$

## Appendix B. Interpretation of Components of the Reproduction Number

To derive the reduction in susceptibility, we computed *S* and $Q_{S}$ at disease-free equilibrium, i.e.,

$$\frac{S^{*}}{N}=\frac{\epsilon }{\epsilon +q},$$

and $Q^{*}=N-S^{*}$. The susceptibility under quarantine is now $(S^{*}+\left(1-\xi \right)Q^{*})/N$. Thus, the difference of susceptibility between quarantine and without quarantine is

$$1-\left(\frac{S^{*}}{N}+\left(1-\xi \right)\frac{Q^{*}}{N}\right)=\frac{\xi q}{\epsilon +q}.$$

To derive the reduction in infectivity due to case detection in the quarantine of exposed individuals, we consider the ratio between the rate of new infections after an exposure period and the rate of outflow from exposed class, i.e.,

$$\frac{\text{Rate of new infections after exposed period}}{\text{Rate of outflow from exposed class}}=\frac{\sigma E+\epsilon Q_{E}}{(q+\sigma )E}.$$

It is easy to see that this ratio becomes unity if there is no detection, i.e, d=0. Otherwise, the rate of outflow from quarantine must be dropped. The difference between these two cases gives the percentage of reduced infectivity. Since we are concentrated on the steady state, the number of exposed individuals in quarantine can be approximated by

$$Q_{E}^{*}=\frac{qE^{*}}{\epsilon +d}.$$

Substituting into the above formula, we have

$$\text{Percentage of reduced infectivity}=\frac{dq}{\left(q+\sigma )(\epsilon +d\right)}.$$

## Appendix C. Estimation of the Number of Primary Cases and Transmissibility in the Second Wave

The number of primary cases and their transmissibility were estimated. We fixed the remaining parameters such as the quarantine rate and its effectiveness by using the same values estimated in the early phase of the epidemic. We also fixed the duration since the primary cases are introduced to the first peak, which accounted for the day that the highest number of cases were reported, i.e., 20 days from the first of December. We observed that the initial guess of the number of primary cases in the optimization may alter the estimation results, while the initial guess of the transmission rate of symptomatic cases has little effect. We performed the estimation several times by randomly choosing them and calculated the error matrix, which was illustrated in Figure A1. The result shows that the minimum error is $2.5\times 10^{-5}$, obtained at the starting point (2,12). At this point, we obtained the estimated asymptomatic and symptomatic primary cases as one and twelve cases, respectively (see Figure A2).
